# Nurses' attitudes towards factors determining the safety of patients treated in pediatric departments

**DOI:** 10.3389/frhs.2025.1648265

**Published:** 2025-09-24

**Authors:** Iwona Malinowska-Lipień, Izabela Sowińska, Sylwia Kocur, Agnieszka Kruszecka-Krówka, Maria Kózka, Agnieszka Gniadek, Łukasz Lompart, Urszula Kalemba, Marta Kasper, Tomasz Brzostek

**Affiliations:** ^1^ Institute of Nursing and Midwifery, Faculty of Health Sciences, Jagiellonian University – Medical College, Krakow, Poland; ^2^ Long-Term Care Facility, Specialist Hospital Ludwik Rydygier in Krakow, Krakow, Poland

**Keywords:** attitudes, hospital, nurses, pediatric department, patient safety

## Abstract

**Introduction:**

Patient safety in healthcare is strongly influenced by safety culture, shaped by organizational beliefs, values, and effective management.

**Material and methods:**

The study involved 434 nurses from the largest pediatric hospital in southern Poland, one of ten single-profile pediatric hospitals in the country. Data were collected using the Polish version of the Safety Attitudes Questionnaire (SAQ-SF) and a sociodemographic survey.

**Results:**

The highest percentage of positive responses (score ≥75) was observed in job satisfaction (56.91%) and stress recognition (53.23%). The lowest results were found in perceptions of management personnel (31.80%) and safety climate (36.41%). Stress levels negatively correlated with the number of nurses per department and shift. Lower assessments of management were associated with higher patient loads and fewer staff. Better working conditions were positively correlated with higher staffing levels.

**Conclusions:**

Nurses in pediatric departments reported high job satisfaction and awareness of stress but low ratings of management and safety climate. Higher nurse staffing levels were associated with lower reported stress, indicating a relationship between staffing levels, work environment, and perceptions of patient safety.

## Introduction

Patient safety depends on many factors in healthcare, including the quality of care provided and the effectiveness of management. It is crucial not only to provide an appropriate level of care, but also to take care of maintaining the health of patients and preventing health problems. Patient safety culture encompasses a system of values, attitudes, competencies, and behavioural patterns, both individual and group, that shape the commitment to and management of a healthy and safe organization (1, 2).

Over the past few decades, patient safety has become one of the most important health priorities worldwide, serving as one of the primary goals of healthcare institutions (3). Despite the focus on safety policy to improve the quality of care, the occurrence of adverse events has increased significantly, especially in hospital settings. Adverse events related to nursing care are a major cause of morbidity and mortality worldwide. They have a significant impact on the healthcare sector, harming patients, but also increasing the cost of care and reducing the credibility of the institution (4).

Healthcare workers, including nurses, physicians, and paramedics, play a key role in strictly following procedures, applying good practices, and providing information needed for continuous improvement (5). It is important for each medical entity to regularly analyse its strengths and weaknesses in terms of patient safety culture, which can especially help hospitals in identifying current problems related to patient safety (6). It is assumed that a healthcare system that implements a patient safety culture actually translates it into actions that reduce the number of adverse events and the resulting costs (6). The assessment of safety culture in a hospital allows for the identification of factors related to the work process that may affect patient safety.

Currently, there is a growing interest in the occurrence of medical errors in the paediatric environment. Children require healthcare tailored to their specific needs, which often requires spending more time on care and performing medical procedures. For this reason, children should be treated in facilities focused on optimizing the safety and well-being of children, both in terms of equipment and personnel trained in paediatric care (7, 8).

Two decades ago, the seminal report To Err Is Human: Building a Safer Health System highlighted that suboptimal health care delivery systems and poorly designed processes significantly contribute to patient safety incidents. In pediatric departments, subsequent research and collaborative safety initiatives have shown measurable improvements in reducing serious safety events; however, persistent contributory factors, such as limited situational awareness, underscore the ongoing need for targeted interventions to reinforce nurses' roles in safeguarding patient safety (9). In this context, highly reliable organizations—characterized by complex environments, where errors can have serious consequences but occur infrequently—provide a valuable model for pediatric hospitals, although implementing consistently reliable and sustainable patient care processes across all clinical settings remains a significant challenge (10).

The studies presented here are important not only from the point of view of patient safety, but also due to the improvement of the quality of services provided in paediatric facilities. Paediatric departments are a relatively under-research area. Most studies focus on factors that determine the safety of adult patients during hospitalization (8). For this reason, it is necessary to develop research focused on the specific needs of children in order to effectively raise healthcare standards in this area. The aim of this study was to assess the patient safety culture in paediatric hospital departments from the perspective of healthcare professionals.

## Material and methods

### Study organization

The study was conducted between September 2023 and February 2024 among nurses employed in the largest paediatric hospital in the Lesser Poland voivodeship. It is one of 10 single-profile pediatric hospitals in Poland. In the hospital, the coordinator appointed by the hospital management was responsible for conducting the study, who was responsible for cooperation with the research team and for ensuring the correctness of data collection in accordance with the study guidelines. The survey questionnaire and envelope were delivered to nurses by the research coordinator. Participants had 6 weeks to complete the questionnaire. Completed questionnaires, packed in an envelope, were placed in a box with holes. The boxes were located in each hospital department. After 6 weeks, the coordinator was responsible for collecting the boxes, securing them and handing them over to the research team. Participants were informed that participation was voluntary and anonymous, that all responses would be confidential and that individual responses would not be available to the hospital management.

The inclusion criterion for the study was: 1/consent to participate in the study; 2/employment as a nurse for at least one month at the time of the study. The exclusion criterion was: 1/ other hospital employees; 2/ lack of consent to the study; 3/ nurses on maternity leave, long-term sick leave or sabbatical leave. The size of the trial was calculated using the method of covariance structure modeling (11), and the minimum required sample for our study was 377. The minimum group size was calculated based on the total population of 21,175 nurses registered with the Malopolska Region Chamber of Nurses and Midwives as of 1 September 2023, assuming an estimated fraction size (p) of 50%, a significance level (α) of 5% (0.05), and a permissible margin of error (e) of 5%. A total of 800 questionnaires were distributed, of which 447 were returned, which was a 55.9% return rate. 434 questionnaires were included in the final analysis, rejecting more than 10% of those not completed on the SAQ (Safety Attitudes Questionnaire Short Form) scale.

All study participants were of Polish nationality and got their vocational education in Poland.

### Ethical considerations

The study was conducted with the consent of the Bioethics Committee of the Jagiellonian University no. 1072.6120.241.2022.

### Research tools

The study used a diagnostic survey method using the SAQ questionnaire in the Polish version adapted by Malinowska-Lipień et al. (Safety Attitudes Questionnaire—SAQ-SF PL) and an original survey questionnaire (12). The SAQ questionnaire is used to assess attitudes towards factors determining patient safety. SAQ-SF is a commonly used tool to assess the attitudes of healthcare workers towards the issue of safety in the workplace and patient safety. Numerous studies indicate that SAQ has good psychometric properties. Translations into fourteen languages (including Albanian, Arabic, Danish, Chinese, Croatian, Dutch, German, Italian, Norwegian, Polish, Portuguese, Slovenian, Swedish, and Turkish) indicate that the psychometric properties are stable (12, 13). He reliability of the Safety Attitude Questionnaire (SAQ) measured using Cronbach's alpha coefficient was 0.98. Before conducting the validity analysis of the Polish adaptation of the SAQ-SF, the Kaiser test was used to check whether the data met the requirements of factor analysis. The Kaiser-Mayer-Olkin (KMO) value, which is a measure of sampling adequacy, was estimated at 0.87 (df = 8630. *p* < 0.001). This model explained 68% of the total variance of the analysed set of variables (9). The SAQ questionnaire consisted of 41 items, divided into two parts, the first one containing 36 questions divided into six subscales, the second one containing 5 questions regarding the sociodemographic data of the participants. The first part included the subscales: 1/Teamwork climate (TC) (questions 1 to 6), which assesses the perception of the quality of cooperation between employees; 2/Safety climate (SC) (questions from 7 to 13)—assesses the perception of employees' organizational commitment to safety; 3/Job satisfaction (JS) (questions from 15 to 19)—assesses the subjective positive feelings associated with work experience; 4/Stress recognition (SR) (questions from 20 to 23)—assessment of the impact of stressors on work performance; 5/Perception of management (PM) assessed separately at the level of the department and the hospital (questions from 24 to 28) and 6/Work conditions (WC) (questions from 29 to 32), which concern the quality of environmental and logistical support in the workplace (e.g., equipment, supplies and professionals). The first part of the questionnaire contains five questions that are not included in any of the subscales, i.e.,: question 14 regarding the assessment of the manager in terms of ensuring safety and questions from 33 to 36 regarding the assessment of conflicts and cooperation between members of the interdisciplinary team, i.e., nurses, doctors, pharmacists. The respondents answered using a 5-point Likert scale. When calculating the results, a conversion to a 100-point scale was used. The final result ranges from 0 to 100. where 0 means the worst and 100 the best perception of the safety climate. Results equal to or higher than 75 points are considered positive (13).

The original questionnaire consisted of 13 questions regarding sociodemographic and professional data such as age, gender, education, form of employment, additional professional qualifications, total work experience and in the paediatric department the type of shift system in which the surveyed persons work, as well as the number of staff and contracted beds in the department.

### Statistical analysis

The analysis was performed using *TIBCO STATISTICA* 13.3 software package (*StatSoft*, Inc., Tulsa, OK, USA). Descriptive statistics methods were used to present the results obtained on a nominal and ordinal scale, i.e.,: number (n) and percentage (%). In order to present the results obtained in a quantitative scale, descriptive statistics method were used, i.e.,: arithmetic mean (M), median (Me), standard deviation (SD). For each respondent, mean results were calculated separately in each SAQ subscale. The analysis of the significance of differences between mean values in the compared groups was conducted in accordance with the applicable principles of statistical test selection. For this purpose, the distribution of the quantitative variables studied was assessed using the Shapiro–Wilk and Kolmogorov–Smirnov tests. The Mann–Whitney test was used to assess the difference between two groups, while the Kruskal–Wallis ANOVA rank test was used to assess the differences between multiple groups. The determination of the relationship between variables was determined using Spearman's rank correlation. In all analyses, effects for which the probability value *p* was lower than the assumed significance level of 0.05 (*p* < 0.05) were considered significant.

## Results

In the study group of 434 nurses, women constituted 97.70% (*n* = 424). One third of the respondents (33.18%; *n* = 144) were aged 51–60. The smallest group consisted of the oldest people, i.e., over 60 years of age (2.07%, *n* = 9) and the youngest, i.e., between 21 and 30 years of age (15.21%, *n* = 66). More than half of the nurses surveyed has a higher education with a master's degree (51.61%, *n* = 224). The smallest group consisted of people with the shortest overall work experience, i.e., less than 1 year (2.30%, *n* = 10). The largest group had work experience between 21 and 30 years (33.18%, *n* = 144). Some of the respondents, despite having a longer work experience as nurses, had a shorter work experience in the paediatric department, as 7.60% (*n* = 33) had worked for less than a year. The largest percentage of nurses employed in paediatric departments, i.e., 28.12% (*n* = 122), had worked for 11–20 years. Nursing staff working in a 12-hour shift system (day/night) constituted 90.78% of the respondents (*n* = 394). Of the respondents, 36.73% (*n* = 155) had completed specialization, and only 6.40% (*n* = 27) had no additional qualifications. More than half of the respondents (52.76%, *n* = 229) worked in departments with 21–30 beds; Table 1.

**Table 1:** Characteristics of the study group.

Sociodemographic and professional data	*n*	%
Gender		
Woman	424	97.70
Man	10	2.30
Age		
21–30 years	66	15.21
31–40 years	87	20.05
41–50 years	128	29.49
51–60 years	144	33.18
Over 60 years	9	2.07
Education		
Master's degree	224	51.61
Bachelor's degree	89	20.51
Secondary medical	121	27.88
Work experience		
Up to 1 year	10	2.30
1–10 years	92	21.20
11–20 years	74	17.05
21–30 years	144	33.18
Over 30 years	114	26.27
Professional experience in the paediatric department:		
Up to 1 year	33	7.60
1–10 years	116	26.73
11–20 years	122	28.12
21–30 years	108	24.88
Over 30 years	55	12.67
Type of work system		
Day system (7.35 h on working days)	40	9.22
Shift system (day/night 12 h each)	394	90.78
Additional qualifications^a^		
Completed specialist course	113	26.78
Completed qualification course	113	26.78
Completed specialization	155	36.73
During specialization	14	3.32
I do not have additional qualifications	27	6.40
Number of beds in the department		
1–10 beds	26	5.99
11–20 beds	149	34.33
21–30 beds	229	52.76
More than 30 beds	30	6.91

*n* number of respondents.

^a^ Results do not add up to 100% due to the possibility of selecting more than one answer.

In the departments where the nurses studied were employed, the average number of hospitalized patients was 21.3 (Me = 22), with the minimum number being 6 and the maximum reaching 81. The average number of nurses employed in the departments was 32.4 (Me = 22), while the number of physicians was 14.0 (Me = 10). The medical personnel varied depending on the time of day and type of personnel. The largest share of personnel was made up of nurses, both on day and night shifts, while the number of paramedics and medical caregivers was minimal. The average number of nurses on day shifts was 6.7 (Me = 5) and on night shifts 5.8 (Me = 4); Table 2.

**Table 2:** Medical personnel staffing.

Medical personnel staffing	Me	M	Min	Max	SD
Number of patients in the department:	22	21.3	6	81	6.72
Number of medical personnel employed in the department:					
Number of nurses	22	32.4	2	80	20.29
Number of physicians	10	14.0	0	60	10.71
Number of paramedics	0	1.5	0	15	4.12
Number of medical caregivers	0	1.4	0	6	1.86
Day shift medical personnel staffing:					
Number of nurses	5	6.7	2	24	4.21
Number of paramedics	0	0.4	0	9	1.36
Number of medical caregivers	1	0.7	0	3	0.73
Night shift medical personnel staffing:					
Number of nurses	4	5.8	0	16	4.14
Number of paramedics	0	0.4	0	9	1.35
Number of medical caregivers	0	0.1	0	2	0.35

Me, median; M, arithmetic mean; Min, minimum; Max, maximum; SD, standard deviation.

The average score from all domains of the SAQ questionnaire was below the expected value of 75 indicating patient safety. The highest percentage of nurses presented a positive attitude (score ≥75) in the scope of job satisfaction (JS) (56.91%) and stress recognition (SR) (53.23%), while the lowest percentage referred to the assessment of management personnel (PM) and safety climate (SC) (31.80% and 36.41%). In the case of all six subscales, except for the safety climate (SC) and the assessment of the management personnel (PM) in the group of nurses, positive attitudes were presented by more than 40% of the respondents. The nurses surveyed in general achieved the highest average results in the scope of job satisfaction (JS)—73.11. while the lowest assessments were given to the management personnel—the hospital management (PM)—62.93 and the head of the department (PM)—66.03; Table 3.

**Table 3:** SAQ item descriptions and subscale results for SAQ among nurses (*n* = 434).

SAQ subscales		SAQ-A item descriptions			Subscale results for SAQ			
		Percent of disagree	Percent of neutral	Percent of agree	Percent <75	Percent ≥75	Total M ± SD	Me
Teamwork climate	1. Nurse input is well received in this clinical area.	7.38	17.05	74.88	56.45	43.55	68.77 ± 17.83	70.83
	2. In this clinical area, it is difficult to speak up if I perceive a problem with patient care.	27.65	18.66	53.69				
	3. Disagreements in this clinical area are resolved appropriately (i.e., not *who* is right, but *what* is best for the patient).	18.20	25.35	56.45				
	4. I have the support I need from other personnel to care for patients.	10.14	14.29	75.58				
	5. It is easy for personnel here to ask questions when there is something that they do not understand.	6.91	12.44	80.65				
	6. The physicians and nurses here work together as a well-coordinated team.	21.66	27.65	50.69				
Safety climate	7. I would feel safe being treated here as a patient.	10.37	29.26	60.37	63.59	36.41	66.51 ± 17.26	67.86
	8. Medical errors are handled appropriately in this clinical area.	11.52	23.50	64.98				
	9. I know the proper channels to direct questions regarding patient safety in this clinical area.	8.53	13.36	78.11				
	10. I receive appropriate feedback about my performance.	10.83	25.12	64.06				
	11. In this clinical area, it is difficult to discuss errors.	31.57	28.80	39.63				
	12. I am encouraged by my colleagues to report any patient safety concerns I may have.	15.67	30.41	53.92				
	13. The culture in this clinical area makes it easy to learn from the errors of others.	12.44	34.79	52.76				
Job satisfaction	15. I like my job.	4.38	5.99	89.63	43.09	56.91	73.11 ± 19.78	75.00
	16. Working here is like being part of a large family.	15.90	28.11	55.99				
	17. This is a good place to work.	9.22	18.66	72.12				
	18. I am proud to work in this clinical area.	7.14	20.74	72.12				
	19. Morale in this clinical area is high.	11.06	28.57	60.37				
Stress recognition	20. When my workload becomes excessive, my performance is impaired.	16.13	13.59	70.28	46.77	53.23	70.54 ± 23.72	75.00
	21. I am less effective at work when fatigued.	15.90	11.75	72.35				
	22. I am more likely to make errors in tense or hostile situations.	8.53	14.52	76.96				
	23. Fatigue impairs my performance during emergency situations (e.g., emergency resuscitation, seizure).	25.12	19.59	55.30				
Perceptions of management	24. Management supports my daily efforts: Unit/Mgt	14.29	42.17	43.55	58.76	41.24	66.03 ± 22.58	65.00
	24. Management supports my daily efforts: Hosp/Mgt	18.20	51.84	29.95	68.20	31.80	62.93 ± 20.35	60.00
	25. Management doesn't knowingly compromise Pt safety: Unit/Mgt	8.29	29.26	62.44				
	25.Management doesn't knowingly compromise Pt safety: Hosp/ Mgt	6.45	37.79	55.76				
	26. Management is doing a good job: Unit/Mgt	9.22	34.10	56.68				
	26. Management is doing a good job: Hosp/Mgt	7.37	42.40	50.23				
	27. Problem personnel are dealt with constructively by our: Unit/Mgt	14.29	40.32	45.39				
	27. Problem personnel are dealt with constructively by our: Hosp/Mgt	10.83	48.62	40.55				
	28. I get adequate, timely info about events that might affect my work, from: Unit/Mgt	10.60	30.88	58.53				
	28. I get adequate, timely info about events that might affect my work, from: Hosp/Mgt	7.37	39.63	53.00				
Work conditions	29. The levels of staffing in this clinical area are sufficient to handle the number of patients.	26.50	15.21	58.29	56.45	43.55	67.25 ± 18.94	68.75
	30. This hospital does a good job of training new personnel.	10.37	27.88	61.75				
	31. All the necessary information for diagnostic and therapeutic decisions is routinely available to me.	11.52	88.48	0.00				
	32. Trainees in my discipline are adequately supervised.	6.45	26.73	66.82				
Q14.33–36	14. My suggestions about safety would be acted upon if I expressed them to management.	18.66	29.26	52.07				
	33. I experience good collaboration with nurses in this clinical area.	6.22	10.37	83.41				
	34. I experience good collaboration with staff physicians in this clinical area.	14.29	18.89	66.82				
	35. I experience good collaboration with pharmacists in this clinical area.	3.46	47.70	48.85				
	36. Communication breakdowns that lead to delays in delivery of care are common.	28.57	31.57	39.86				

*Hosp, hospital; Mgt, management, Pt, patient; Me, median; M, arithmetic mean; SD, standard deviation; ≥75, positive result; <75, negative result.

The analysis showed that the majority of respondents (80.65%) had no difficulty in asking questions in case of doubts or lack of knowledge. The respondents were aware of who to direct questions regarding patient safety to (78.11%), and indicated that in situations of tension and hostility, the risk of making a mistake increases (76.96%). The majority of respondents (89.63%) declared job satisfaction and indicated good cooperation with other nursing personnel (83.41%) and medical personnel (66.82%); Table 3.

The Teamwork Climate (TC) assessment showed a positive correlation with the Safety Climate (SC) assessment, Job Satisfaction (JS), the assessment of the management personnel at the level of the department manager (PM), the assessment of the management personnel at the level of the hospital management (PM) and with the assessment of Work conditions (WC). Similarly, the assessment of Safety Climate (SC) significantly positively correlated with Job Satisfaction (JS), the assessment of the Management Personnel (Department Manager) (PM), the assessment of the Management Personnel (Hospital Management) (PM) and the assessment of Work conditions (WC). Job satisfaction (JS) significantly positively correlated with the assessment of the Management Personnel (Department Manager) (PM), the assessment of the Management Personnel (Hospital Management) (PM) and Work conditions (WC). Moreover, the assessment of the management personnel at the level of the head of the department (PM) significantly positively correlated with the assessment of the management personnel (hospital management) (PM) and with the assessment of Work conditions (WC). The management personnel (hospital management) assessment (PM) significantly positively correlated with Work conditions (WC); *p* < 0.001; Table 4.

**Table 4:** Correlation matrix for the safety attitudes questionnaire (SAQ) subscales.

SAQ subscales	Corelations (Pearson's r)						
	1	2	3	4	5	6	7
Teamwork climate (TC)	1						
Safety climate (SC)	**0**.74**	1					
Job satisfaction (JS)	**0**.63**	**0**.65**	1				
Stress recognition (SR)	−0.00	−0.04	−0.08	1			
Management personnel assessment (Manager) (PM)	**0**.42**	**0**.44**	**0**.43**	0.01	1		
Management personnel assessment (Management) (PM)	**0**.50**	**0**.52**	**0**.50**	−0.02	**0.12** **	1	
Work conditions (WC)	**0**.44**	**0**.42**	**0**.46**	−0.00	**0.31** **	**0.44** **	1

Bold value indicates statistical significance.

** Correlation is significant *p* < 0.001.

The analysis showed a negative correlation between the stress diagnosis (SR) and the number of nurses employed in the paediatric department, as well as the number of nurses present on day and night shift. A negative correlation was also found between the assessment of the management personnel (PM) and the number of patients under care and the number of nurses employed in the paediatric department. In addition, the analysis showed a positive correlation between the assessment of work conditions (WC) and the number of nurses employed in the paediatric department and the number of nurses on day and night shifts; Table 5.

**Table 5:** The influence of the number of patients and nursing personnel on the attitudes of nurses in paediatric departments.

SAQ subscales	Corelations (Pearson's r)			
	Number of patients	Number of nurses employed in the paediatric department	Number of nurses on day shifts	Number of nurses on night shifts
Teamwork climate (TC)	0.04	−0.06	−0.06	−0.00
Safety climate (SC)	0.02	−0.08	−0.08	−0.01
Job satisfaction (JS)	0.08	−0.01	−0.03	0.05
Stress recognition (SR)	−0.02	**−0**.15*	**−0**.12*	**−0**.18**
Management personnel assessment (Manager) (PM)	0.08	0.02	0.02	0.05
Management personnel assessment (Director) (PM)	**−0**.10*	**−0**.10*	−0.09	−0.06
Work conditions (WC)	0.01	**0**.13*	**0**.14*	**0**.15**

Bold value indicates statistical significance.

* Correlation is significant *p* < 0.05.

** Correlation is significant *p* < 0.001.

The analysis showed statistically significant differences in the assessment of the patient safety level in the subscales of the SAQ questionnaire such as Teamwork Climate (TC), Safety Climate (SC) and Job Satisfaction (JS) SAQ between women and men; *p* < 0.05. The results in these subscales were significantly higher in the female group. No statistically significant differences were found in the assessment of the patient safety level in the individual subscales of the SAQ questionnaire between the age groups of the respondents; *p* > 0.05. A statistically significantly lower assessment of the patient safety level was demonstrated in the subscales: Teamwork Climate (TC) and Safety Climate (SC) in the group of nurses with a master's degree compared to nurses with a bachelor's degree or secondary medical education, *p* < 0.05. The overall work experience of the nurses surveyed did not have a statistically significant effect on the assessment of patient safety in the subscales of the SAQ questionnaire. The analysis showed that the assessment of teamwork climate (TC) was dependent on experience in paediatric departments (*p* < 0.05). The type of shift work system had a significant effect on the assessments in the Teamwork Climate (TC), Safety Climate (SC) and Job Satisfaction (JS) subscales. Personnel working in a single-shift system obtained higher assessments compared to those employed in a shift system; *p* < 0.000; Table 6.

**Table 6:** Comparison of SAQ mean scores across demographic factors (*N* = 434).

Group	Teamwork climate (TC)			Safety climate (S.C.)			Job satisfaction (JS)			Stress recognition (SR)			Perception of management (manager) (PM)			Perception of management (director) (PM)			Work conditions (WC)		
	Me	M	SD	Me	M	SD	Me	M	SD	Me	M	SD	Me	M	SD	Me	M	SD	Me	M	SD
Gender																					
Women (*N* = 424)	70.83	69.18	17.52	67.86	67.00	16.86	75.00	73.54	19.45	75.00	70.40	23.56	65.00	66.03	22.74	60.00	63.14	20.21	68.75	67.35	19.08
Men (*N* = 10)	54.17	51.25	22.91	51.79	45.71	21.68	57.50	55.00	26.03	84.38	76.25	31.01	62.50	66.00	14.87	50.00	54.00	25.14	59.38	63.13	11.20
*p*	**0.008**			**0.002**			**0.012**			0.206			0.985			0.193			0.336		
Age																					
21–30 years (*N* = 66)	70.83	67.55	19.84	64.29	63.96	18.79	75.00	68.94	22.02	75.00	70.93	23.58	65.00	63.86	29.35	60.00	59.55	24.33	68.75	67.32	18.33
31–40 years (*N* = 87)	70.83	66.86	17.25	64.29	65.89	17.45	75.00	70.92	19.73	75.00	72.70	23.52	65.00	65.92	23.90	55.00	61.32	22.65	68.75	68.46	18.81
41–50 years (*N* = 128)	72.92	71.16	18.60	67.86	68.25	18.17	80.00	75.94	19.58	75.00	71.39	24.85	65.00	67.11	22.54	65.00	65.35	20.01	68.75	65.15	20.77
51–60 years (*N* = 144)	66.67	67.97	16.60	64.29	65.77	15.43	75.00	73.26	18.93	75.00	68.36	23.35	65.00	66.32	18.09	65.00	63.54	16.62	75.00	67.46	19.49
>60 years (*N* = 9)	75.00	75.00	14.13	82.14	78.17	14.45	80.00	82.22	12.02	68.75	69.44	17.24	65.00	62.78	21.52	55.00	58.89	23.29	62.50	62.50	11.27
*P*	0.249			0.162			0.221			0.693			0.947			0.746			0.553		
Education level																					
Master of nursing (*N* = 224)	68.75	67.75	15.86	64.29	65.08	16.65	75.00	72.12	18.58	75.00	72.41	22.12	65.00	66.07	22.86	60.00	62.28	19.54	68.75	67.86	17.92
Bachelor of nursing (*N* = 89)	70.83	66.20	22.76	64.29	65.01	20.20	75.00	71.35	23.63	68.75	66.08	27.48	65.00	64.38	25.18	60.00	63.20	24.97	68.75	65.03	21.36
Secondary medical (*N* = 121)	70.83	72.56	16.71	71.43	70.25	15.50	75.00	76.24	18.62	75.00	70.35	23.38	65.00	67.15	20.00	60.00	63.93	18.03	68.75	67.77	18.94
*P*	**0**.**034**			**0**.**015**			0.139			0.290			0.873			0.674			0.575		
Work experience																					
Up to 1 year (*N* = 10)	79.17	75.83	18.92	75.00	70.71	21.61	77.50	75.00	20.68	75.00	71.25	17.97	65.00	60.50	37.75	62.50	65.00	31.45	75.00	73.13	25.52
1–10 years (*N* = 92)	70.83	68.16	19.63	64.29	64.48	18.83	75.00	69.89	21.66	75.00	72.35	24.52	67.50	66.74	28.20	60.00	60.27	22.79	68.75	65.42	19.97
11–20 years (*N* = 74)	70.83	66.10	17.74	64.29	64.62	17.97	75.00	71.28	20.99	75.00	71.03	23.78	65.00	68.04	22.80	60.00	59.93	23.37	68.75	67.57	17.85
21–30 years (*N* = 144)	70.83	70.95	17.94	69.64	69.00	16.73	80.00	76.01	18.64	75.00	71.18	24.45	65.00	65.83	20.86	60.00	66.32	18.04	68.75	68.40	18.55
Over 30 years (*N* = 114)	66.67	67.62	15.90	64.29	65.85	15.49	75.00	73.07	18.49	68.75	67.87	22.68	65.00	64.87	17.49	65.00	62.54	17.25	68.75	66.56	18.78
*p*	0.060			0.088			0.292			0.310			0.972			0.868			0.877		
Professional experience working in a paediatric department:																					
Up to 1 year (*N* = 33)	75.00	71.59	18.96	67.86	67.10	18.98	75.00	68.03	20.00	75.00	75.76	19.50	75.00	66.97	32.09	60.00	59.85	27.77	68.75	65.53	22.38
1–10 years (*N* = 116)	70.83	68.86	20.92	67.86	66.29	20.34	75.00	71.29	23.70	75.00	70.15	25.67	65.00	64.57	25.39	60.00	63.19	23.70	68.75	67.73	20.31
11–20 years (*N* = 122)	66.67	66.12	16.55	64.29	65.08	15.88	75.00	73.36	17.88	75.00	70.70	23.32	70.00	67.87	19.94	60.00	61.35	18.87	68.75	68.44	16.07
21–30 years (*N* = 108)	70.83	71.10	15.24	67.86	68.32	15.54	80.00	76.94	17.20	75.00	69.79	23.30	70.00	66.99	20.74	65.00	65.65	16.49	68.75	67.42	17.74
Over 30 years (*N* = 55)	66.67	68.18	17.30	64.29	66.23	15.44	75.00	71.91	18.67	75.00	69.32	23.88	65.00	62.55	18.28	60.00	62.36	17.40	68.75	64.32	22.04
*p*	**0**.**018**			0.129			0.054			0.685			0.099			0.959			0.796		
Type of work system																					
Day system (*N* = 40)	79.17	75.94	19.42	80.36	75.89	19.03	90.00	83.00	17.79	80.36	75.89	19.03	75.00	71.38	25.79	70.00	67.00	19.11	71.88	72.03	17.39
Shift system (*N* = 394)	70.83	68.04	17.53	64.29	65.55	16.80	75.00	72.11	19.72	64.29	65.55	16.80	65.00	65.48	22.19	60.00	62.51	20.44	68.75	66.77	19.05
*p*	**0**.**005**			**0**.**000**			**0**.**000**			0.417			0.075			0.214			0.166		

Bold value indicates statistical significance.

Me, median; M, arithmetic mean; SD, standard deviation; *p*- value.

## Discussion

Caring for a child during hospitalization requires taking into account not only their health needs, but also their developmental specificity and family context when planning services. Regardless of the reason for hospitalization and clinical factors, the priority of child care is always to ensure broadly understood safety. A nurse, due to the professional tasks performed, is a person who is particularly responsible for creating a positive hospitalization climate and patient safety at every stage of the hospital stay. The assessment of factors determining the safety of a paediatric patient from the perspective of healthcare providers—nurses, is therefore the basis for optimizing activities in this area. Studies conducted in several countries have shown high rates of medication errors in paediatric patients, ranging from 41.8% to 72% (14–16). Meanwhile, Khan et al. also found that the rate of medical errors and preventable adverse events in hospitalized children was 6.0 per 100 admissions and 1.8 per 100 admissions, respectively. Medical errors caused paediatric patients to stay in hospital longer and were more likely to suffer from metabolic or neuromuscular disorders (17).

In the current study, most of the subscales related to selected factors determining the safety of hospitalized children were negatively assessed by respondents, similarly to the studies by Alquwez et al. and Hessels et al. (18, 19). The lowest score was for management personnel and safety climate, and positive attitudes were demonstrated only in the area of personal job satisfaction and stress recognition. As indicated by the authors of other studies, nurses' job satisfaction is related, among other factors, to the support they receive from their superiors (15, 16). According to Parry et al., positive attitudes of the personnel towards patient safety concern areas related to independent care, such as job satisfaction, sense of safety at work, working conditions and perception of the role of management. In turn, areas of interdependent care, such as teamwork climate and stress management, receive moderate and negative assessments (20). Similar conclusions were presented by Brasaitė (21). The low score for the assessment of management personnel and safety climate obtained in our own study suggests that these areas constitute a significant challenge in the context of improving the working conditions. Contemporary studies conducted in various research centres clearly indicate a significant impact of the quality of leadership on the level of satisfaction and well-being of medical personnel (22). Insufficient support from the management personnel can lead to a decreased sense of justice in the organization, which in turn lowers job satisfaction and increases stress levels among employees. The lack of appropriate safety procedures or their insufficient respect due to limited awareness of medical teams (insufficient number of trainings/mediocre quality of trainings) can lead to a sense of unsafe work, which has a negative impact on the general well-being of team members and their commitment to the reliable performance of their duties (23). The results of the study presented by Mears et al. confirm the positive relationship between nurses' engagement and safety culture in the workplace (24), which is consistent with the results of the current study on paediatric departments. In view of the above, an important goal of the management team's actions should be to gain the trust and respect of employees through transparent operations based on a system of clear procedures.

In the current study, a significant predictor of higher job satisfaction was single-shift work, which partially corresponds to the results of other authors (25). Kaya points to the multifactorial conditioning of the level of nurses' job satisfaction, emphasizing the importance of, among others, age, work experience, and level of their education. In the cited report, a higher level of job satisfaction was achieved in the group of nurses with longer work experience (11–15 years), with a bachelor's and specialist's degree and working in a single-shift system than among nurses with short work experience, working in a shift system (25). However, Torun et al. (26) obtained different results, according to which the determinant of nurses' job satisfaction was primarily the specificity of the department. In view of the differences in the results of the own study and those presented by other authors, it seems reasonable to expand this aspect in planning further studies in the area of professional satisfaction of nurses.

A study of 350 nurses working in a University hospital in Egypt found that the education level and experience were the main factors associated with attitudes towards patient safety, while gender had no significant association with attitudes (27). Compared with less experienced nurses, experienced nurses have a higher attitude toward patient safety (28, 29). This finding was similar to that of Al-Mugheed et al. (30) who found that younger nurses had lower safety attitude scores than those with more experience. Professional characteristics of nurses, including education and experience, as well as nursing systems that address staffing levels, influence the quality of care they provide. For example, it is believed that a higher percentage of registered nurses (RN) with a bachelor's degree in hospitals will help in the effective detection and prevention of adverse events because these nurses will have greater knowledge, more effective communication skills, and the ability to monitor patients (31).

Compared to men, the women surveyed rated the level of patient safety higher in the subscales of Teamwork climate, Safety climate and Job satisfaction, similarly to the reports of Kakemam et al. (6). Khoshakhlagh et al. (32) obtained different results. It is indicated that women in the nursing profession more often experience higher levels of job satisfaction and a better perception of the atmosphere at work, which may result from their role in nursing teams, which are often characterized by greater communication and cooperation (32). However, healthcare units, and especially hospitals, with a strong safety culture, are characterized by communication based on trust, a common understanding of the importance of safety, and faith in the effectiveness of preventive measures. These systems, by establishing norms of behaviour, help ensure patient safety (32). Employees of these organizations, regardless of gender and other socio-demographic variables, have positive beliefs about the functioning of internal systems that support cooperation between work units and organizational structures. On the other hand, it should also be noted that the studies did not take into account cultural factors, including the perception of the role of women in a given community, as well as systemic factors, which could also determine the differences in the presented results.

It was shown that the age of the nurses surveyed did not differentiate their assessment of patient safety during hospitalization. A statistically significantly lower result of the patient safety level in the Teamwork Climate and Safety Climate subscales was obtained in the group of nurses with a master's degree compared to nurses with higher vocational education or secondary medical education. In turn, other reports have yielded varied results in this respect. Khoshakhlagh et al. did not find any significant differences between the patient safety culture score and personnel age (32), while according to Kakemam et al., nurses with less than 1 year of experience had a better perception of patient safety than nurses with longer experience (22). Yin et al. indicated that nurses under 25 years of age obtained higher scores in terms of attitudes related to patient safety than nurses in other age categories (33). Younger nurses have less professional experience, which may determine a lower awareness of the child's health, social and developmental needs/threats during hospitalization, and at the same time determine lower requirements for ensuring patient safety. On the other hand, in the modern system of nurse education, the aspect of patient safety and prevention of broadly understood iatrogenesis is clearly emphasized, which certainly shapes the attitudes of students and later graduates. Perhaps it was the variable contribution of these factors that contributed to the divergent results of our own study and the presented reports.

A relationship has been shown between the number of nurses employed in the paediatric department and the staffing on day and night shifts, and the attitude towards recognizing stress among the personnel. The optimal number of employees, especially in the context of working in departments requiring specialist care, such as paediatric departments, is crucial for ensuring a high level of services and work comfort. It enables an even division of duties, reduces the sense of pressure and increases the time to provide high-quality care (18). In the study by Hessels et al., it was indicated that higher patient safety culture scores were associated with fewer personnel shortages in nursing care (19). Moreover, Khoshakhlagh et al. and Yin et al. showed that in the opinion of medical personnel, shift work had a negative impact on ensuring the safety of children during hospitalization. Nurses whose weekly working hours were higher also had a lower sense of patient safety culture compared to nurses who worked fewer hours per week (32, 33). In the current study, shift personnel performing 12-hour day/night shifts constituted over 90% of respondents. It is emphasized that irregular and frequent night shifts and working overtime cause overload in nurses and disrupt their circadian rhythm. Thus, they have a negative impact on the health and bio-psycho-social functioning of nurses (33), which may affect the professional tasks they perform and result in the occurrence of adverse events and broadly understood iatrogenesis. Therefore, the basis for optimizing care in the context of children's safety during hospitalization should be the absolute adjustment of the number of personnel on duty to the established standards taking into account the specificity of the department, as well as reasonable work planning aimed at using the potential of employees on the one hand and protecting against psycho-physical overload on the other. Scientific reports indicate that the appropriate number of personnel in the nursing team directly translates not only into the quality of patient care, but also into job satisfaction and mental health of medical personnel (19).

An important result of this study is the indication of a positive correlation between the assessment of the teamwork climate, safety climate and job satisfaction, assessment of management personnel and working conditions. This indicates the need for an integrated approach to nursing personnel management, which will primarily include ensuring better working conditions, optimizing relationships between members of the interdisciplinary team and improving the effectiveness of the management staff.

An integrated approach, taking into account all these aspects, can contribute to an increased sense of safety at work, which has a direct impact on the job satisfaction of nurses. Contemporary research highlights that employees who feel supported by their superiors and have access to appropriate resources and training demonstrate higher levels of engagement and job satisfaction (22). Good practices in personnel management, such as proper staffing, regular performance appraisals, feedback, transparency of management activities and investment in employee professional development, are crucial in building a positive organizational climate. In addition, promoting teamwork and communication, especially in the context of teamwork, contributes to creating a more effective work environment, which in turn reduces stress levels, improves the quality of care provided, and increases the level of safety of patients (23). This is particularly important in the context of childcare, due to the specific developmental characteristics that determine various health, developmental and social needs.

### Limitation of the study

The study is limited by its single-centre nature, as the analysis was conducted only in one paediatric hospital in southern Poland, which limits the possibility of generalizing the results to other facilities. In addition, potential confusing factors, such as workload or the specificity of individual paediatric departments, which could have influenced the results but were not analysed in detail, were not taken into account. Because the study had a cross-sectional design, causal relationships cannot be established; this should be explicitly acknowledged as a limitation. Therefore, in subsequent studies, it is planned to extent the scope of the analysis to other paediatric hospitals in Poland and include additional groups of medical personnel, such as physicians and paramedics, which will allow for a more comprehensive picture of patient safety.

## Conclusions

1. Nurses in pediatric departments reported high job satisfaction and strong awareness of stress but low ratings of management personnel and safety climate.
2. Higher nurse staffing, particularly with coverage across day and night shifts, was associated with lower reported stress levels.
3. Correlations were observed between teamwork climate, safety climate, job satisfaction, management assessment, and working conditions.

## Data Availability

The raw data supporting the conclusions of this article will be made available by the authors, without undue reservation.
